# Effects of *Gymnema inodorum* Leaf Extract on the Alteration of Blood Coagulation Parameters and Platelet Count in *Plasmodium berghei*-Infected Mice

**DOI:** 10.1155/2022/4225682

**Published:** 2022-03-11

**Authors:** Kongsak Boonyapranai, Orawan Sarakul, Voravuth Somsak, Suriyan Sukati

**Affiliations:** ^1^Research Institute for Health Sciences, Chiang Mai University, Chiang Mai 50200, Thailand; ^2^School of Allied Health Sciences, Walailak University, Nakhon Si Thammarat 80161, Thailand; ^3^Research Excellence Center for Innovation and Health Products, Walailak University, Nakhon Si Thammarat 80161, Thailand

## Abstract

Malaria remains highly prevalent and one of the major causes of morbidity and mortality in tropical and subtropical regions. Alteration of blood coagulation and platelets has played an important role and attributed to increased morbidity in malaria. Hence, this study was performed to investigate the efficacy of *Gymnema inodorum* leaf extract on *Plasmodium berghei*-induced alteration of blood coagulation parameters and platelet numbers in mice. Groups of ICR mice were inoculated with 1 × 10^7^ parasitized red blood cells of *P. berghei* ANKA (PbANKA) and given orally by gavage with 100, 250, and 500 mg/kg of *G. inodorum* leaf extract (GIE). Chloroquine (10 mg/kg) was used as a positive control. Platelet count and blood coagulation parameters were measured. The results showed that PbANKA induced thrombocytopenia in mice as indicated by markedly decreased platelet count. Decreased platelet count had a negative correlation with the degree of parasitemia with *R*^2^ value of 0.6668. Moreover, significantly (*p* < 0.05) shortened activated partial thromboplastin time was found in PbANKA-infected group, while prothrombin time and thrombin time were still normal. GIE gave significantly (*p* < 0.05) good results with respect to platelet count, compared with the results obtained from positive and healthy controls. Additionally, GIE reversed the alteration of blood coagulation parameters when compared to untreated mice. The highest efficacy of GIE was observed at a dose of 500 mg/kg. It was concluded that GIE exerted a protective effect on thrombocytopenia and altered blood coagulation parameters induced by PbANKA infection in mice. This plant may be a future candidate for alternative antimalarial development.

## 1. Introduction

Malaria is a parasitic disease caused by protozoa in the genus *Plasmodium* and transmitted by the female *Anopheles* mosquito. Five species of malaria parasites that have been well known to cause human malaria include *Plasmodium falciparum*, *Plasmodium vivax*, *Plasmodium malariae*, *Plasmodium ovale*, and *Plasmodium knowlesi* [1]. Globally, an estimated 229 million malaria cases and 409,000 malaria deaths were reported in 2019. The highest malaria burden is centered in Africa, with children aged less than five years being the most affected [2]. The emergence and resurgence of *Plasmodium* parasite resistance to several available antimalarial drugs have been hindering the control and eradication of malaria [3]. The death of malaria is caused by systemic complications such as cerebral malaria, severe hemolytic anemia, hypoglycemia, metabolic acidosis, acute kidney injury, respiratory distress, liver damage, hematological complications, and alterations biochemical parameters [4–6]. Thrombocytopenia and coagulation disorders are involved in the development of severe malaria [7, 8]. These problems have prompted researchers attempting to search and find new antimalarial substances for alternative malarial treatment, especially from natural products such as plant extracts [9]. Research on plant extracts against malaria has increased due to the evidence of their pharmacological potential [10]. For instance, quinine and artemisinin isolated from *Cinchona ledgeriana* and *Artemisia annua* have been shown the potent antimalarial activity and developed as standard drugs for malaria treatment [11].

*Gymnema sylvestre*, which belongs to the family of Apocynaceae, is a wild herb found in India, Africa, Australia, and China. It is traditionally used to treat various diseases, including diabetes, malaria, dyspepsia, constipation, jaundice, hemorrhoids, renal and vesicle calculi, cardiopathy, asthma, bronchitis, amenorrhea, and leukoderma [12]. Several researches revealed pharmacological potentials, including antidiabetic, hypoglycemic, antioxidant, anti-inflammatory, anticancer, immunosuppressive, hepatoprotective, and anti-infectious activities [13]. The important chemical substances in *G. sylvestre* extract have also been reported, such as phenols, flavonoids, quinones, tannins, saponins, anthraquinones, triterpenoids, and gymnemic acids [13]. *Gymnema inodorum* (Lour.) Decne, also a member of the genus *Gymnema*, is popular in Thailand, especially in the northern part. *G. inodorum* leaf extract has been used as a traditional food and medicine in Asia [14]. It has been described that *G. inodorum* leaf extract showed the inhibition of glucose absorption with high antioxidant activity [15]. *Gymnema* is also traditionally used in wound healing and having a good wound healing property in rat model [16, 17]. However, the effect of *G. inodorum* leaf extract on blood coagulation disorders in mice has not yet been studied. Hence, this study focused on evaluating the effects of *G. inodorum* leaf extract on blood coagulation parameters and platelets in *Plasmodium berghei*-infected mice.

## 2. Materials and Methods

### 2.1. Plant Collection and Authentication

The leaves of *Gymnema inodorum* were collected from Chiangda organic garden, Chiang Mai, Thailand, in October 2020. Identification and authentication of the collected plant were conducted at the Research Institute for Health Sciences, Chiang Mai University, and the voucher specimen (NRU64/036-001) was deposited.

### 2.2. Preparation of Aqueous Extract

The fresh leaves of *G. inodorum* were cleaned and dried in a hot air oven at 50°C overnight. The dried plant was ground into a coarse powder with mortar and pestle and subsequently prepared to found powder using an electric blender. 5 g of the plant material was extracted in 100 ml distilled water (DW) at a 60°C incubator with an occasional shaker for 15 min. After centrifugation at 2,500 rpm for 15 min, the supernatant was collected, and the residue was reextracted for a second time by adding another DW. The supernatant was combined and concentrated using lyophilization to obtain the aqueous crude extract of *G. inodorum* leaves (GIE). Finally, the GIE was stored in a desiccator until use [18].

### 2.3. Experimental Mice

Pathogen-free male ICR mice aged 6-8 weeks, weighing 25-35 g purchased from Nomura Siam International Co., Ltd., were used. Mice were acclimatized for 7 days before being used for the experiments. They were kept at 22-25°C, 12 h light-12 h dark cycle, with a pellet diet and clean drinking water *ad libitum*. All animal experiments with care and handling were conducted according to the NIH guidelines. Ethical approval was obtained from the animal ethics committee, Walailak University (WU-AICUC-63-031).

### 2.4. Parasite

The chloroquine-sensitive *Plasmodium berghei* strain ANKA (PbANKA) was obtained from the MR4 (Malaria Research and Reference Reagent Resource Center). The parasite was maintained by intraperitoneal serial passage of infected blood from mouse to mouse. The infected donor mouse with a rising parasitemia of 20-30% was sacrificed, and the blood was collected by cardiac puncture into a vacuum tube containing heparin as an anticoagulant. The blood was then diluted by phosphate-buffered saline (PBS) based on the parasitemia of the donor mouse and the number of red blood cells (RBC) from the normal mouse (5 × 10^9^ RBC/ml) in such a way that 1 ml of blood contained 5 × 10^7^ parasitized RBC. Naïve mouse was subsequently inoculated intraperitoneally with 0.2 ml of infected blood containing 1 × 10^7^ parasitized RBC of PbANKA.

### 2.5. Measurement of Parasitemia

Parasitemia was determined by microscopic examination of Giemsa-stained blood smear. Tail blood of each mouse was collected, and a thin smear on microscopic slides was prepared. After being allowed to air-dry, the smeared slides were fixed with absolute methanol and stained with 10% Giemsa at room temperature for 10 min. The stained slides were viewed microscopically using a light microscope with a 100x objective. The parasitemia was estimated by counting the number of parasitized RBC out of RBC in random 5 fields with approximately 200-500 cells. The percentage of parasitemia was calculated using the following formula:

(1) $$\begin{array}{c} \%\text{Parasitemia}=\frac{\text{Number of parasitized RBC}\times 100}{\text{Total number of RBC}}. \end{array}$$

### 2.6. Measurement of Blood Coagulation Parameters and Platelet Numbers

Mouse blood was collected by cardiac puncture into a tube containing 3.2% buffered sodium citrate and K_3_EDTA for coagulation tests and platelet count, respectively. Platelet count was done using an automated blood count (Mindray BC-5180, Shenzhen, China). Platelet-poor plasma was prepared from citrate blood by centrifugation at 3000 × *g* for 15 min and immediately frozen at -80°C until use. Blood coagulation tests, including prothrombin time (PT), activated partial thromboplastin time (APTT), and thrombin time (TT), were measured with a semiautomated blood coagulation analyzer (HumaClot Duo Plus, Wiesbaden, Germany) using human reagents. The experiments were performed according to the manufacturer's instructions in duplicate.

### 2.7. In Vivo Efficacy Assay of GIE

To determine the effect of GIE on the alteration of blood coagulation parameters, Peter's 4-day test was used in PbANKA-infected mice [19]. Naïve ICR mice randomly grouped with the equal number of 3 mice in each group were used. On the first day (D0) of the experiment, mice were inoculated intraperitoneally with the standard inoculum of PbANKA (1 × 10^7^ parasitized RBC). After three-hour postinfection, the three groups were administered orally by gavage with 100, 250, and 500 mg/kg of GIE by dissolving in 0.2 ml of DW once a day for 4 consecutive days (D0-D3). The other two groups were carried out as negative and positive controls, which were given 0.2 ml of DW and 10 mg/kg of chloroquine (CQ) dissolved in DW, respectively. Additionally, normal mice were also used as healthy controls. On D4, blood was collected by cardiac puncture for measuring platelet count and blood coagulation parameters, including PT, APTT, and TT.

### 2.8. Statistical Analysis

GraphPad Prism version 6.0 (GraphPad Software Inc., CA, USA) was used for analysis in this study. A one-way ANOVA followed by Tukey's posttest was employed to compare the parameters between the control and extract-treated groups at D4 of the study. All results were expressed by the mean ± standard error of mean (SEM), and statistical significance was considered if *p* < 0.05 at the 95% confidence interval.

## 3. Results

### 3.1. Thrombocytopenia Induced by PbANKA Infection in Mice

**Figure 1:**
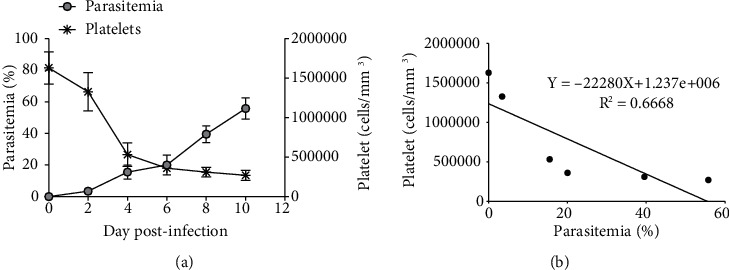
PbANKA-induced thrombocytopenia. ICR mice were infected with 1 × 10^7^ parasitized RBC of PbANKA by intraperitoneal injection. (a) Parasitemia and platelet count were monitored. (b) Correlation between parasitemia and platelet count was also observed. Results were presented as mean ± SEM.

As shown in Figure 1(a), PbANKA-associated thrombocytopenia in mice was observed. Parasitemia was detectable from D1 postinfection with parasitemia less than 1% and reached 55.7 ± 3.9% on D10. Moreover, PbANKA resulted in thrombocytopenia as indicated by the progressive decrease of platelets, which occurs when ascending parasitemia from D4-D10 postinfection. Additionally, a negative correlation (*R*^2^ = 0.6668) between parasitemia and platelet count was also found.

### 3.2. Antithrombocytopenia of GIE in PbANKA-Infected Mice

**Figure 2:**
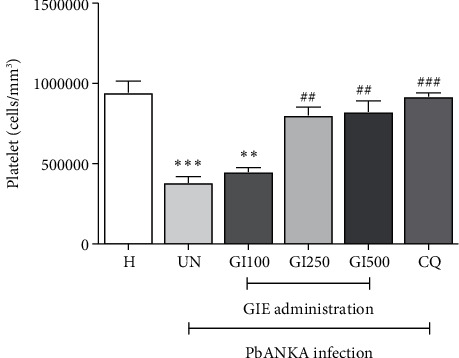
Antithrombocytopenia effect of GIE on PbANKA-infected mice. Groups of ICR mice were inoculated with 1 × 10^7^ parasitized RBC by intraperitoneal injection. GIE (100, 250, and 500 mg/kg) was given orally by gavage for 4 consecutive days. Platelet count was measured using an automated analyzer. The results were presented as mean ± SEM. H: healthy; UN: untreated; GI100, GI250, and GI500: GIE treatment at doses of 100, 250, and 500 mg/kg; CQ: 10 mg/kg of chloroquine. ^∗∗^p < 0.01 and ^∗∗∗^p < 0.001 compared to H. ^##^p < 0.01 and ^###^p < 0.001 compared to UN.

GIE showed antithrombocytopenia induced by PbANKA infection in mice (Figure 2). PbANKA induced thrombocytopenia as indicated by a significant (*p* < 0.001) decrease in platelet count in the untreated group compared to healthy controls. Interestingly, in GIE- (250 and 500 mg/kg) treated mice, platelet count was restored to a normal level compared to healthy controls. There were no statistically significant differences between 250 and 500 mg/kg of GIE- and CQ-treated groups. However, GIE at a dose of 100 mg/kg did not present antithrombocytopenia during PbANKA infection as indicated by a significant (*p* < 0.01) decrease of platelet count compared to healthy controls.

### 3.3. Effects of GIE on Blood Coagulation Parameters during PbANKA Infection

**Figure 3:**
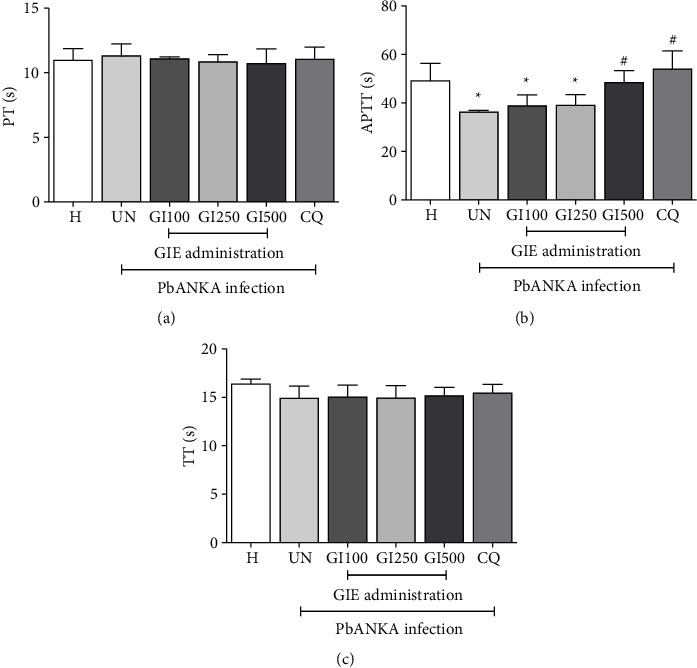
Effect of GIE on blood coagulation parameters in PbANKA-infected mice. Groups of ICR mice were inoculated with 1 × 10^7^ parasitized RBC by intraperitoneal injection. GIE (100, 250, and 500 mg/kg) was given orally by gavage for 4 consecutive days. Blood coagulation parameters, including (a) PT, (b) APTT, and (c) TT, were measured using an automated analyzer. The results were presented as mean ± SEM. H: healthy; UN: untreated; GI100, GI250, and GI500: GIE treatment at doses of 100, 250, and 500 mg/kg; CQ: 10 mg/kg of chloroquine. ^∗∗^p < 0.01 compared to H. ^#^p < 0.05 compared to UN.

To evaluate the effect of GIE on blood coagulation parameters during PbANKA infection, PT, APTT, and TT were investigated. As shown in Figure 3, PbANKA did not cause prolonged PT and TT in untreated groups. Surprisingly, significantly (*p* < 0.05) shortened APTT was observed in untreated and GIE- (at the doses of 100 and 250 mg/kg) treated mice compared to healthy control. However, APTT was normalized compared to healthy controls in 500 mg/kg of GIE- and CQ-treated groups.

## 4. Discussion

Hematological abnormalities have been observed in patients with malaria as being the most common [20]. Significance of thrombocytopenia and blood coagulation disorders and their relevance in acute and severe malaria has also been reported [7, 21]. They are a hallmark of blood-stage malaria infections, which are related to increased parasite propagation. The results of PbANKA infection in mice revealed that thrombocytopenia was observed as indicated by markedly decreased platelet counts. This finding was consistent with several studies that have shown malaria-associated thrombocytopenia [22–24]. Possible causes of thrombocytopenia during malaria include reduced platelet survival from peripheral destruction, enhanced sequestration, and decreased platelet production [25, 26]. It has been described that thrombocytopenia could lead to increased replication of parasites by either decreased parasite killing or decreasing activation of the immune response, as shown by the negative correlation between parasitemia and platelet count [27, 28]. However, there is still considerable uncertainty about the role of platelets in malaria. Activation of the liver to release acute phase proteins or pathogenic immune response by platelet has also been reported [29]. Platelet and erythrocyte sequestration are frequent in the severe forms of malaria, and thrombocytopenia is present [30]. Thrombocytopenia in this study might also be associated with endothelial damage and isolated platelet consumption [31]. Our results showed significant increases in platelet count in mice treated with 250 and 500 mg/kg of GIE and CQ when compared to healthy control, suggesting their role as an acute phase reactant to infection. However, 100 mg/kg of GIE may be considered to have a lower effect on thrombocytopenia induced by PbANKA infection. It could be due to active substances at low levels resulting in the activity may not be detected at a low dose.

Significant alteration of APTT in PbANKA infection was observed in our study. It could be due to the hepatic involvement associated with malaria infection. Liver damage induced by PbANKA infection in mice has been reported with a strong association to hemostatic changes [32, 33]. Moreover, the systemic inflammatory response to malaria can influence the coagulation system. Tumor necrosis factor- (TNF-) *α* and interleukin- (IL-) 10 have the greatest impact on the inflammation during malaria infection, followed by thrombocytopenia and blood coagulation disorder [34–36]. The results showed that the alteration of APTT in malaria was reversed by administration with 500 mg/kg of GIE. It might be due to the antioxidant and anti-inflammatory activities of GIE. In addition, the hepatoprotective effect of GIE in PbANKA infection could also be considered. However, 100 and 250 mg/kg of GIE did not present the protective effect on shortened APTT in PbANKA infection in mice.

## 5. Conclusion

The results obtained from this study revealed that GIE had protective effects against thrombocytopenia during PbANKA infection in mice at the doses of 250 and 500 mg/kg. Moreover, the effect on restored blood coagulation parameters of GIE has also been observed. The significant effects of GIE accompanied by its relative safety may confirm the traditional use of GIE. However, the identification and characterization of active compounds and its mechanisms should be further determined whether these compounds have good activity to be considered good candidates for the development of alternative antimalarial drugs.

## Data Availability

The figure data used to support the findings of this study have been deposited in https://figshare.com/s/8682d4dbf7fce1b4f4b9 repository (DOI 10.6084/m9.figshare.14371952).
